# Ten simple rules for writing a Registered Report

**DOI:** 10.1371/journal.pcbi.1010571

**Published:** 2022-10-27

**Authors:** Emma L. Henderson, Christopher D. Chambers

**Affiliations:** 1 School of Psychology, University of Surrey, Guildford, Surrey, United Kingdom; 2 Cardiff University Brain Research Imaging Centre, School of Psychology, Cardiff, United Kingdom

## Introduction

Registered Reports are an increasingly popular publishing format that is currently offered in more than 300 journals. Because the process of writing and submitting a Registered Report is different to that of standard manuscripts, we felt it important to create this “10 Simple Rules” guide for writing a more open and useful manuscript.

## What are Registered Reports?

Registered Reports are a form of research article where the study protocol is reviewed before the study is undertaken. They are designed to reduce publication bias and various forms of reporting bias by using a 2-stage writing and peer review process. Before the research is conducted, authors submit a Stage 1 manuscript that includes an introduction (with hypotheses where relevant) and detailed methods and analysis plans. Following peer review and revision, the decision to publish is made based on evaluation of the research question and the rigour of the methods, and is therefore results-agnostic. If the article is accepted, authors receive an “in-principle acceptance” that commits the journal or platform to *publishing the final research regardless of the outcome*. Authors then conduct their research as outlined in their Stage 1 and complete a Stage 2 manuscript in which the results and discussion sections are added to the approved Stage 1 protocol. The completed manuscript undergoes a second round of peer review focusing on compliance with the Stage 1 plans and assessing whether the conclusions are valid given the results. Following possible revisions, the Registered Report is published.

### Benefits of Registered Reports

Registered Reports bring a wealth of benefits both for the research community and for the individual researcher. A key benefit is that they provide a powerful antidote for publication bias, see [1,2]: The decision to publish is results-agnostic because it is taken pre-study and results-blind. This principle not only ensures that both “positive” and “negative” results are equally likely to be published, but also guarantees publication independent of outcome (as long as you follow your Stage 1 plans, see rule 8) while releasing the pressure on authors to present “positive,” ground-breaking, or novel results [3]. Thus, this format not only alleviates the aforementioned biases, but also the stress on researchers navigating their way through a “publish or perish” culture.

Receiving the commitment to publish your research before the study is run (in-principle acceptance at Stage 1) means that you can add the paper to your CV as a concrete output (i.e., you can include the in-principle acceptance date and journal/platform) much earlier than for a standard article where you have to wait until the study is completed and accepted. This is particularly vital for early career researchers (ECRs) applying for their first jobs and grants and is perhaps among the reasons why the majority of published Registered Reports are first authored by ECRs [3].

A further salient benefit for the individual researcher is that Stage 1 peer review occurs when it matters most—before any primary research is conducted (in some cases, pilot work may have been completed) and at a time when the authors can improve the quality of their research by adjusting their plans.

### Types of Registered Reports

To date, the majority of the over 700 (at time of publication) published Registered Reports report confirmatory, experimental work. However, as the format develops, Registered Reports are increasingly being used for more diverse types of research. The following formats currently exist (note not all journals that offer Registered Reports support all formats, so check your target journal early, see rule 3):

**Confirmatory:** So called “primary Registered Reports” report hypothesis-testing (confirmatory) research using newly generated data and currently make up the bulk of published Registered Reports. These papers may include a single study (e.g., [4]) or several prespecified studies (e.g., [5]).

**Existing data:** So called “secondary Registered Reports” use data that already exist to answer a research question (e.g., [6]). If there is a potential risk of bias because the data have already been observed, you will need to address this risk (see here for further information, including a level-based taxonomy of bias control for Registered Reports involving existing data).

**Meta-analyses, systematic reviews, and systematic maps:** Protocols for research synthesis studies are often publicly registered, but the Registered Reports format has the added benefits of protocol peer review and in-principle acceptance (e.g., systematic review and meta-analysis: [7]; systematic map: [8]).

**Qualitative**: Many aspects of qualitative research can be specified a priori (e.g., [9]). Authors can refer to guidance on qualitative preregistration [10].

**Incremental:** You can add a new study to an accepted Registered Report. This option is appropriate where studies are interdependent, for example, the results of study 1 inform the design of study 2, or where an important exploratory finding warrants a second study within the same article (more information here, no examples at the time of publication).

**Programmatic:** For larger or longer-term projects, programmatic Registered Reports offer the option to publish several Stage 2 manuscripts from a single approved Stage 1 (e.g., [11]).

The rules below detail practical recommendations to help researchers with both experimental and non-experimental research. Rules 1 to 6 relate to the steps leading up to Stage 1 in-principle acceptance and 7 to 10 to post acceptance.

## Rule 1: Learn on the job

Use the period of writing your Stage 1 manuscript to learn before you conduct your study, so that when you come to run it, you have already anticipated potential pitfalls and know how you will handle, analyse, and interpret your data. Starting work on your Registered Report from as early a stage as possible will help guide your focus and learning. Most journals or platforms that offer Registered Reports have clear guidelines on their statistical (e.g., conducting a statistical sampling plan) and methodological requirements, some of which you may not be familiar with. Knowing the parameters against which your work will be judged *before* you design it allows you to learn the right things at the right time or to seek out collaborators with appropriate expertise as necessary. Ensure your collaborators are familiar with the Registered Reports format. All coauthors should understand the primary aim of Registered Reports to reduce bias, which requires critical design and analysis decisions to be made before conducting the research, and the Stage 1 manuscript to remain largely unchanged in the final paper. For introductory guides to Registered Reports, see [3,12].

The format also front-loads important decisions to the start of the research project, when you’re motivated and excited about the study. You will receive reviewer feedback when it’s most useful—before you start your research—allowing you to improve the design in ways that would be impossible had the study already been run as with traditional peer review. So when you start data collection (or analysis in the case of secondary data), you will have everything ready to complete your study, including a detailed, peer-reviewed study protocol.

## Rule 2: Develop an empirically valid question and a sound, feasible study design that can answer that question

Without an empirically valid question, you do not have the basis of a Registered Report: The philosophy behind Registered Reports is that what gives research its value is the question being asked and the quality of the methods used, not the results. Valid research questions are usually derived from theory, applications, or gaps in knowledge. At Stage 1 peer review, the editor and reviewers will evaluate the empirical validity of your research question(s), and some journals may also assess its subjective importance. You should use your introduction to explain why the question needs to be answered and how the study will be informative regardless of the outcome (e.g., whether the hypothesis is supported or not). To do this, you should describe the logic and rationale for your research question(s); your hypotheses (where applicable) should follow directly from your research question(s), be precisely stated, and translate theoretical predictions into observable outcomes.

The second key criteria reviewers assess at Stage 1 is the soundness and feasibility of your methodology and analysis plan to test your questions. In terms of soundness, you should consider design features that maximise the rigour and informativeness of your study (regardless of outcome) such as sample size, blinding, randomisation, participant recruitment criteria, prespecification and justification of inclusion and exclusion criteria, validity (see [13]), generalisability (see [14]), and outcome-neutral checks (also known as “control checks,” “positive controls,” “manipulation checks,” “tests of intervention fidelity,” or “sanity checks”) that confirm that the study is sufficiently well designed to be capable of answering the research questions. Outcome-neutral checks test the auxiliary assumptions in your design, for example that your independent variable manipulates what it intends to, by targeting a variable (other than the dependent variable of interest) that the independent variable would be expected to influence. Such checks show you and reviewers that the study worked as intended, and a “negative” result therefore cannot be ascribed to a failed manipulation. For more information, see [15].

In the event of a failed outcome-neutral check, the study may still be informative in showing that a procedure does not perform as intended, perhaps even challenging the status of an assumed reality check [15]. In such cases, the commitment to publishing the Stage 2 manuscript is likely to be maintained provided there are additional indicators that the study was undertaken to a sufficiently high standard. In rare, severe cases, where outcome-neutral checks and all other critical quality checks fail, the article may be rejected at Stage 2. However, a more likely outcome in that case is that authors would be given the opportunity to redesign the study. This would be treated as an incremental registration in which the authors add a study to the approved submission, and the new study undergoes Stage 1 peer review.

For Registered Reports involving hypothesis testing, reviewers are evaluating the extent to which your study minimises false positives (i.e., incorrectly concluding that an effect exists) and false negatives (i.e., incorrectly concluding that there is no effect). Again, these factors maximise the informativeness of the study regardless of outcome. Prespecifying your analysis plan constrains researcher degrees of freedom and helps minimise false positives. You should prespecify your sampling plan (e.g., statistical power analysis) for each hypothesis including the reasoning for your effect size, the rationale for any specified statistical priors, cut offs, collapsing analyses across groups, etc. You should also consider designing your analysis plan to maximise the informativeness of null results by using equivalence testing [16] or Bayesian analyses to support claims of invariance between conditions [17].

Pilot data, though not a requirement of Registered Reports, is especially useful to test and show reviewers that your planned design is feasible (pilot data is not typically useful for calculating power analyses as it introduces bias; see [18] for an explanation and [19] for recommendations). As well as allowing you to check feasibility, pilot data may reveal unanticipated exclusion criteria for example, and will give you data to plan your data analysis steps in order to write your Stage 1 protocol and code (code is preferable to narrative explanations because it is more precise). If you are writing a meta-analysis, systematic review, or systematic map, you should pilot your searches to ensure that you will have sufficient studies included to provide a meaningful answer to your research question(s). This level of planning is one of the benefits of Registered Reports over vanilla preregistration; you do not just specify the topline design, but also detail all the processes and steps behind that design, so you won’t end up wedded to a design that is infeasible in practice. Any research conducted prior to your Stage 1 submission should be noted as such (e.g., “All steps in this search term identification section were completed prior to submitting the Stage 1 Registered Report”).

The template in S1 Appendix provides further information on the criteria necessary for designing a rigorous Registered Report.

## Rule 3: Select your journal (or don’t) and understand the journal requirements while planning your study

While developing your study, plan where you’re going to submit your work. There are 2 main options: submit to a single journal that offers Registered Reports or to the supra-journal platform Peer Community In Registered Reports (PCI RR).

For the former option, refer to the current list of journals that offer Registered Reports. Beyond checking the disciplinary scope of your target journal, also check that the journal accepts the type of study that you’re designing. All journals that currently offer Registered Reports accept confirmatory hypothesis testing research and a subset accept qualitative studies, systematic reviews, or meta-analyses as Registered Reports.

The alternative option is submitting to the free, supra-journal platform PCI RR (https://rr.peercommunityin.org/), where Registered Reports from any research field are reviewed and accepted as preprints. Once accepted, the reviews and editorial recommendation are published on the PCI RR website and authors have the choice to keep their Stage 2 article as a peer-reviewed Registered Report preprint (with DOI) or to submit it as a Registered Report to one of several “PCI RR-friendly journals” that have committed to publishing Registered Reports accepted by PCI RR without further peer review. That is, when you receive in-principle acceptance from PCI RR, you automatically receive in-principle acceptance from all eligible journals. PCI RR accepts a wide range of Registered Reports including quantitative and qualitative studies, systematic reviews, systematic maps, scoping reviews, and meta-analyses.

Check the author guidelines of your intended outlet for more details. Identify submission requirements (e.g., any minimum requirements for power or Bayes Factors, requirements to openly share data) and ensure that you can adhere to them with your design and resources. If you’re initially submitting to PCI RR but ultimately have a journal planned for publication, you’ll need to make sure you comply with your intended journal’s conditions for Registered Reports submissions. These do differ among PCI RR-friendly journals, so make sure you plan ahead.

As with standard manuscripts, you may wish to send the editor a pre-submission enquiry to check the suitability of your manuscript for the journal. However, unlike standard manuscripts, you could also use this enquiry to discuss any study-specific concerns, for example, any constraints you have on the timing of data collection.

## Rule 4: Consider when to apply for ethics

**Fig 1:**
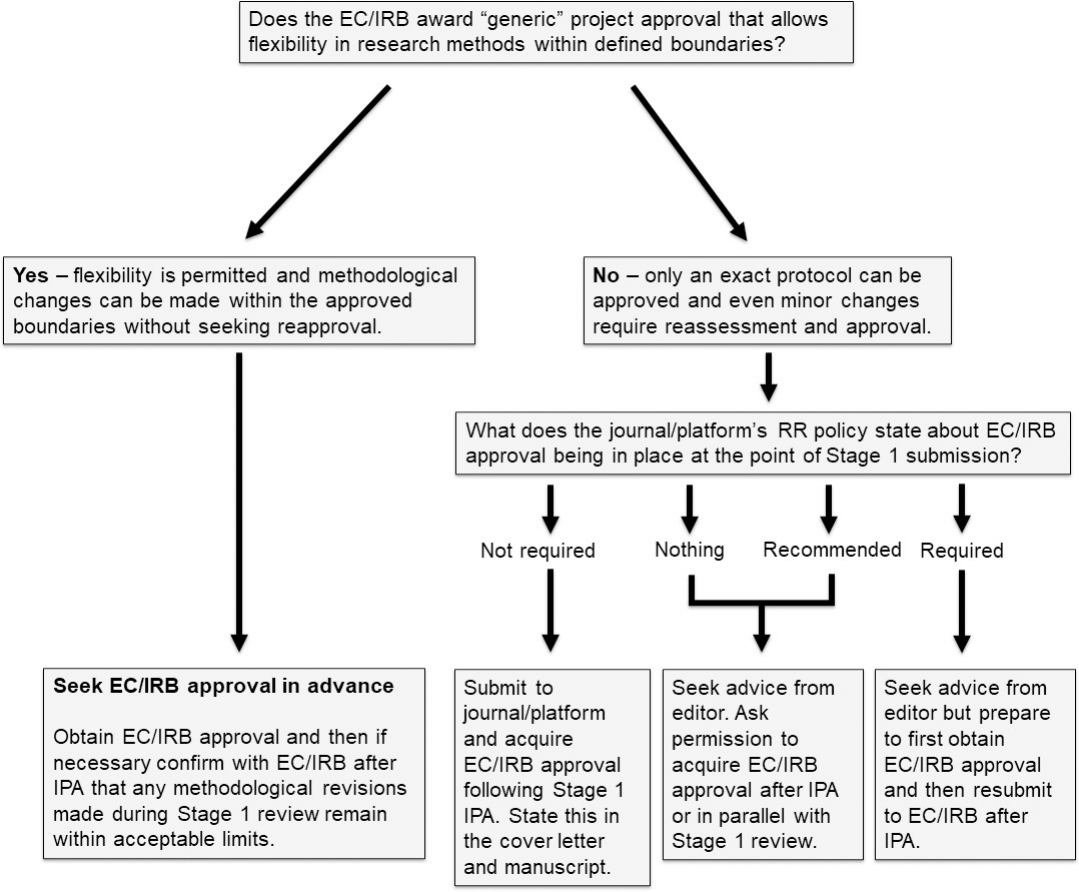
Registered Reports Ethics Flowchart. EC, ethics committee; IPA, in-principle acceptance; IRB, institutional review board.

If your research requires ethical approval from your institution, you will need to consider when to apply for it (see Fig 1). Check both your target outlet’s policy regarding ethical approval and also your institution’s policy on accepting amendments or allowing flexibility for minor deviations. If the journal or platform requires ethical approval at Stage 1 submission and your institution allows some flexibility, obtain ethical approval prior to Stage 1 submission and then once you have received in-principle acceptance, check with your review board that any changes are within the limits of their flexibility. If no flexibility is allowed, and your study plans have changed as a result of the peer review process, you’ll need to resubmit your ethics once you have your in-principle acceptance and a firm study plan.

When you submit your Stage 1 Registered Report, you will typically be asked to provide an anticipated timescale for your Stage 2 submission. If you need to seek ethical approval after in-principle acceptance, don’t forget to factor this into your timescale.

## Rule 5: Map from research question through to interpretation

In your Stage 1 Registered Report, provide clear links between your research question(s), hypotheses (if applicable), sampling plan (if applicable), analyses, through to interpretation. This linking ensures that any predictions made in the introduction are transparently connected to the analysis in the results section and the conclusions inferred from the pattern of results. There are a couple of ways that you can make sure that these elements are explicitly connected. First, number each research question (Q1, Q2 …) and/or hypothesis (H1, H2 …) and add this suffix to corresponding analyses and interpretations (for an example, see [4]).

Second, an unambiguous way to report this critical information (and this also helps with planning the study) is to create a design summary table listing each research question, hypothesis, sampling plan, the analyses that will test those hypotheses and the results that will confirm or disconfirm each prediction. See S1 Appendix for a blank design summary table and S2 Appendix for examples of completed tables that have been published in peer-reviewed Stage 1 preprints and awarded in-principle acceptance by PCI RR. If your analysis plan depends on the results (e.g., parametric versus nonparametric tests), then specify the contingencies for making different choices using IF-THEN statements.

## Rule 6: Specify what you’ll do and what you won’t do

Registered Reports aim to reduce various forms of reporting bias by eliminating undisclosed flexibility in the procedures and analyses. Your Stage 1 manuscript should therefore be precise and comprehensive in its level of detail; an independent researcher in your field should be able to replicate your research without seeking additional information [12]. This level of clarity demands an attention to detail beyond that of standard papers, requiring the inclusion of every procedural specific no matter how mundane or apparently inconsequential [12]. In practice this means, for example, listing the *order* in which exclusion criteria will be applied, as well as details of the exclusions themselves. Pilot data may prove useful here, see rule 2. In addition to specifying what you will do (e.g., “We will contact authors and request details of unpublished studies.”), you should also specify what you will not do (e.g., “If after 2 attempts to contact the authors there is no response, we will not contact them again.”).

To meet the requirement to provide precise and exhaustive detail, the method section of Registered Reports is often longer than those of traditional papers. Journals sometimes avoid imposing word limits for Registered Reports, but where space is limited, use appendices to supplement the main text. Using a design summary table (see rule 5) will help improve the clarity of your analysis plans. Authors of meta-analyses, systematic reviews, or systematic maps should also consult guidelines such as PRISMA [20] and NIRO [21] for the type and level of detail to be included in the Stage 1 manuscript (see https://www.equator-network.org/ for a list of reporting guidelines by study type).

## Rule 7: Preregister your Stage 1 manuscript and conduct your study

At the point you receive in-principle acceptance of your Stage 1 manuscript, you should preregister it, either under embargo (you may wish to embargo your Stage 1 manuscript, for example, to avoid potential participants reading your plans and hypotheses) or publicly, in a public repository like the Open Science Framework. Select “Registered Report Protocol Preregistration”, this is *very brief* and designed specifically for Registered Reports (you do not need to write a separate new preregistration). It asks for details of the in-principle acceptance date, journal, and a PDF of your Stage 1 Registered Report and associated materials. You should also include everything that forms part of your approved Stage 1, such as any pilot data and/or simulations and related analyses, and all your materials and code. At some journals/platforms, such as Cortex and PCI RR, the editorial team will preregister the accepted Stage 1 manuscript for you.

Once you have received in-principle acceptance and preregistered, you can conduct your study in the knowledge that it will be published regardless of the outcome. Ensure that you run your study in line with the Stage 1 protocol.

## Rule 8: After Stage 1 approval: Manage any deviations from your Stage 1 manuscript and communicate with the editor

Unanticipated developments or events outside your control may necessitate a change to your methods post-Stage 1 approval (e.g., an additional exclusion criteria, procedural deviation, or technical error). Any such change must be recorded and *transparently reported* in the Stage 2 manuscript as a deviation from the approved protocol (for example, see Table 2 in [4], where non-preregistered exclusion criteria are marked with an asterisk). If the change is substantial (i.e., has the potential to change the type or validity of inferences that can be drawn), you should immediately seek the approval of the editor, who may obtain input from the Stage 1 reviewers. What is deemed substantial will vary based on your research design. Examples include a change to equipment, materials, participant population, inferential analyses, or coding scheme. If you are unsure whether or not to contact the editor, it is better to err on the side of caution and seek editorial approval rather than risk Stage 2 rejection due an unauthorised deviation from protocol. Failure to do so could result in the Stage 2 manuscript being rejected (when reviewers are focused on assessing adherence to the Stage 1 plans). Remind the editor of any approved changes in your Stage 2 cover letter.

You can also transparently document any such changes *as they occur*, either by noting them in a time-stamped, open lab book (for an example see https://osf.io/jcvue/), or in the case of substantial changes, by updating your preregistration. In the Open Science Framework repository, unexpected changes can be appended to the original preregistration by selecting “update” on your original registration.

## Rule 9: Prepare your Stage 2 manuscript

Your introduction and method sections in your Stage 2 paper should not deviate unnecessarily from your approved Stage 1 manuscript, other than changing from future to past tense (e.g., “we will test” changes to “we tested”) or correcting any factual errors or misunderstandings. If pertinent new research is published in the meantime, this should not be added to your introduction because it did not motivate the research. Instead, it can be included in your discussion. Equally should your understanding of the topic evolve post Stage 1 acceptance, this can be added to the discussion. If having such information upfront is *essential* to the understanding of the paper, it can be added to the introduction as a footnote that explicitly states the additional information is a deviation from the accepted Stage 1. You should explain why having this information upfront is essential.

Registered Reports constrain the space for post hoc decisions, therefore for hypothesis-driven studies, all confirmatory (i.e., hypothesis testing) analyses should be included in the analysis plan in the Stage 1 manuscript and must be reported in the final paper (unless, for example, a fatal flaw is detected in the analysis and the omission is agreed with the editor and reviewers: In such cases, the omission should be noted in the final manuscript). At Stage 2, additional exploratory, data-dependent (i.e., hypothesis generating) analyses are welcome provided that they are justified in the text and clearly distinguished as exploratory (i.e., in a section labelled “Exploratory Analyses”). The distinction is critical because the unbounded nature of exploratory research makes it susceptible to undesirable outcomes such as the effects of bias, inflated alpha levels, and low power. Results based on exploratory analyses should be considered tentative and in need of verification via further confirmatory research. When interpreting such results in your discussion, make it clear that your exploratory analyses are generating hypotheses, not testing them.

For non-hypothesis testing research, such as qualitative research, systematic reviews, or systematic maps, all research questions should be defined in the Stage 1 manuscript. Additional questions that arise from the data should be explicitly noted as such.

## Rule 10: Make your Stage 1 Registered Report and study assets open

Like other forms of open research, Registered Reports are about transparency. When your Stage 2 manuscript is accepted, you should make your Stage 1 preregistered manuscript publicly available (many journals/platforms will require you to do so anyway). This will allow readers of your published Stage 2 manuscript to compare the 2 versions. At the same time, you should make your data, code, and materials as open as possible within ethical and legal constraints; indeed, many journals make such transparency a mandatory requirement.

## Conclusion

Registered Reports encourage methodological rigour (see [22]) and transparent planning and reporting, while reducing biases and likely representing a less distorted and selective picture of research than standard papers (see [2,3]). These 10 simple rules provide guidance for writing and submitting a Registered Report. The authors of this paper are also happy to answer questions directly. For more information on Registered Reports, see the central Registered Report hub (https://www.cos.io/initiatives/registered-reports).

## Supporting information

S1 Appendix Registered Report and Design Planner Template. (DOCX)

S2 Appendix Example Completed Design Planner Tables. (DOCX)
