# Supplementary material for: Ten simple rules for writing a Registered Report
Source: PLoS Comput Biol. 2022 Oct 27;18(10):e1010571. doi: 10.1371/journal.pcbi.1010571 (PMC9612468; doi:10.1371/journal.pcbi.1010571)
Supplement: S1 Appendix — (DOCX) [file pcbi.1010571.s001.docx]

S1 Appendix

Registered Report and Design Planner Template

**If you can answer these TEN questions you will have built the engine of a Stage 1 Registered Report**

**1) What is the main question being addressed in your study?**

- *Why is it important that we answer this question? What’s the big picture?*

**2) Describe the key independent and dependent variable(s), specifying how they will be measured.**

- *Ensure that they are defined precisely*

**3) What are your hypotheses?**

- *Ensure that your predictions are defined precisely in terms of the specific IVs and DVs*
- *Listing them as Hypothesis 1, Hypothesis 2 etc (with corresponding H0 in each case, as appropriate) is recommended*

**4) How many and which conditions will participants/samples be assigned to?**

- *Where applicable be sure to include details of randomisation, blinding and counterbalancing. Make it clear whether the design is within-subjects, between-subjects, mixed, or other.*

**5) How many observations will be collected and what rule will you use to terminate data collection?**

- *Ensure that your stopping rule takes into account any data exclusions.*
- *If adopting null hypothesis significance testing, what power will your study achieve? What effect size will you target and why? Remember that you are choosing the smallest effect size of theoretical or applied interest, or the smallest you can feasibly detect. For an actual RR you can use pilot data to help motivate this estimate, but you shouldn’t rely on pilot data alone because it is vulnerable to bias.*
- *If adopting Bayesian sampling methods, what is your prior? And what is your criterion Bayes factor for asserting relative support of H0 or H1, or your maximum resource limit?*

**6) What are your study inclusion criteria?**

- *How will participants/samples be recruited/included and under what specific rules?*

**7) What are your data exclusion criteria?**

- *State rules for excluding data both at the level of samples/participants (within groups) and at the level of raw data (within samples/participants), e.g., conditions involving data quality, completeness and outliers.*
- *Remember to be comprehensive: exclusion criteria are very difficult to change after data collection has commenced because doing so risks introducing bias. Think about previous experiments you have done and all the reasons you have ever thrown out a data set or data point.*

**8) What positive controls or quality checks will confirm that the obtained results are able to provide a fair test of the stated hypothesis?**

- ***WHAT’S THIS?*** *A positive control tests the existence of phenomena that would confirm that the IV, DV or instrumentation was used correctly and is therefore capable of testing the main study predictions. One of the most famous positive control experiments was the use of the* [*Galileo spacecraft to test for the existence of life on Earth*](http://www-pw.physics.uiowa.edu/~dag/publications/1993_ASearchForLifeOnEarthFromTheGalileoSpacecraft_NATURE.pdf)*. If the instrumentation on the probe couldn’t detect life on Earth (i.e. had the positive control failed), then it would not be reasonable to use to the probe to test the hypothesis that life existed on other planets.*
- *Not all experimental designs have suitable positive controls. Where a positive control isn’t possible, think of what quality checks or verifications you would build into your design* ***before results are known*** *to convince a sceptic that you had conducted the experiment to a sufficient standard (e.g., noise within certain limits etc.). Make sure these are independent of your main hypothesis tests.*
- *Where a positive control (e.g., manipulation check) or quality check (e.g. lack of floor or ceiling effects in data) requires a statistical test, ensure that the test is adequately powered or sampled.*

**9) Specify exactly which analyses you will conduct to examine the main question/hypothesis(es)**

- *Ensure that there is an* ***exact*** *correspondence between each scientific hypothesis and each statistical test. Failure to precisely specify these links is one of the main reasons RRs are rejected.*
- *If your analysis strategy will depend on the results (e.g., normal vs. non-normal distribution) then specify the contingencies for making different choices, i.e. IF-THEN statements.*
- *In the event of a negative result, would you be happy to conclude that there “was no evidence of a difference” between conditions, or would you instead want to be able to make the stronger claim that “there is evidence of no difference between conditions”? The first inference is limited to absence of evidence while the second (stronger) one refers to evidence of absence. If you want to make the stronger inference, you will need* [*Bayesian inferential methods*](https://link.springer.com/content/pdf/10.3758%2Fs13423-017-1230-y.pdf) *or* [*frequentist equivalence testing*](http://journals.sagepub.com/doi/pdf/10.1177/1948550617697177)*.*
- *Complete the design planner below to make the links absolutely clear between the research question (or questions), hypothesis (or hypotheses), sampling plans, analysis plans, and contingent interpretation*

| Question | Hypothesis | Sampling plan | Analysis Plan | Rationale for deciding the sensitivity of the test for confirming or disconfirming the hypothesis | Interpretation given different outcomes | Theory that could be shown wrong by the outcomes |
| --- | --- | --- | --- | --- | --- | --- |
|  |  |  |  |  |  |  |
|  |  |  |  |  |  |  |
|  |  |  |  |  |  |  |
|  |  |  |  |  |  |  |

*Design Planner Guidance Notes:*

- ***Question****: Articulate each research question being addressed in one sentence.*
- ***Hypothesis****: Where applicable, a prediction arising from the research question, stated in terms of specific variables rather than concepts. Where the testability of one or more hypotheses depends on the verification of auxiliary assumptions (such as positive controls, tests of intervention fidelity, manipulation checks, or any other quality checks), any tests of such assumptions should be listed as hypotheses. Stage 1 proposals that do not seek to test hypotheses can ignore or delete this column.*
- ***Sampling plan****: For proposals using inferential statistics, the details of the statistical sampling plan for the specific hypothesis (e.g., power analysis, Bayes Factor Design Analysis, ROPE etc). For proposals that do not use inferential statistics, include a description and justification of the sample size.*
- ***Analysis plan****: For hypothesis-driven studies, the specific test(s) that will confirm or disconfirm the hypothesis. For non-hypothesis-driven studies, the test(s) that will answer the research question.*
- ***Rationale for deciding the sensitivity of the test for confirming or disconfirming the hypothesis****: For hypothesis-driven studies that employ inferential statistics, an explanation of how the authors determined a relevant effect size for statistical power analysis, equivalence testing, Bayes factors, or other approach.*
- ***Interpretation given different outcomes****: A prospective interpretation of different potential outcomes, making clear which outcomes would confirm or disconfirm the hypothesis.*
- ***Theory that could be shown wrong by the outcomes****: Where the proposal is testing a theory, make clear what theory could be shown to be wrong, incomplete, or otherwise inadequate by the outcomes of the research.*

**10) Are you proposing to collect new data or analyse existing data?**

- *If the proposal involves existing data, what steps will you take to ensure that your analysis plan isn’t biased by any prior observation you have had of the data?*

***You might be wondering:*** *why is there no section for specifying exploratory analyses? That’s because for RRs we usually don’t allow authors to specify exploratory analyses in Stage 1 submissions. A central strength of the RR format is the unequivocal distinction it draws between confirmatory pre-registered analyses and exploratory unregistered analyses. Pre-specifying (usually vague) plans for exploratory analyses blurs this separation. Any analysis that can be precisely planned should be specified as confirmatory at Stage 1, even if a secondary hypothesis. And any analysis that can’t be precisely planned should be withheld until Stage 2, where it is then introduced and comprehensively reported in the Exploratory Analyses section of the Results.*

**Tips for Avoiding Desk Rejection at Stage 1**

Many Registered Report submissions are desk rejected at Stage 1, prior to in-depth review, for failing to sufficiently meet the Stage 1 editorial criteria. In many such cases, authors are invited to resubmit once specific shortcomings are addressed, although major problems can lead to outright rejection. To help minimize the chances of authors’ submissions being desk rejected, we list below the top ten reasons why Stage 1 submissions are rejected prior to review.

1. Cover letter doesn’t make necessary statements concerning ethics, data archiving, and so forth (check specific author guidelines).
2. The protocol contains insufficient methodological detail to enable replication and prevent researcher degrees of freedom. One commonly neglected area is the criteria for excluding data, both at the level of animals/participants and at the level of data within animals/participants. In the interests of clarity, we recommend listing these criteria systematically rather than presenting them in prose.
3. Lack of correspondence between the scientific hypotheses and the pre-registered statistical tests. This is a common problem and severe cases are likely to be desk rejected outright. To maximize clarity of correspondence between predictions and analyses, authors are encouraged to number their hypotheses in the Introduction and then number the proposed analyses in the Methods to make clear *which analysis tests which prediction*. Ensure also that power analysis, where applicable, is based on the actual test procedures that will be employed to test those hypotheses; e.g. don’t propose a power analysis based on an ANOVA but then suggest a linear mixed effects model to test the hypothesis.
4. Power analysis, where applicable, fails to reach the minimum level stated in journal policy.
5. Power analysis is over-optimistic (e.g., based on previous literature but not taking into account publication bias) or insufficiently justified (e.g., based on a single point estimate from a pilot experiment or previous study). Proposals should be powered to detect the smallest effect that is plausible and of theoretical value. Pilot data can help inform this estimate but is unlikely to form an acceptable basis, alone, for choosing the target effect size.
6. Intention to infer support for the null hypothesis from statistically non-significant results, without proposing use of Bayes factors or frequentist equivalence testing.
7. Inclusion of exploratory analyses in the analysis plan. Manuscripts proposing exploratory analyses will usually be desk rejected until such analyses are removed because inclusion of exploratory “plans” at Stage 1 blurs the line between confirmatory and exploratory outcomes at Stage 2. Instead, such analyses can be included at Stage 2 and need not be pre-registered. Under some circumstances, exploratory analyses could be discussed at Stage 1 where they are necessary to justify study variables or procedures that are included in the design exclusively for exploratory analysis.
8. Failure to clearly distinguish work that has already been done from work that is planned. Where a proposal contains a mixture of pilot work that has already been undertaken and a proposal for work not yet undertaken, authors should use the past tense for pilot work but the future tense for the proposed work. At Stage 2, all descriptions shift to past tense.
9. Lack of pre-specified positive controls or other quality checks, or an appropriate justification for their absence. We recognise that positive controls are not possible with all study designs, in which case authors should discuss why they are not included.
10. Where applicable, lack of power analysis within proposed positive controls that depend on hypothesis testing.
