# Supplementary material for: Ten simple rules for writing a Registered Report
Source: PLoS Comput Biol. 2022 Oct 27;18(10):e1010571. doi: 10.1371/journal.pcbi.1010571 (PMC9612468; doi:10.1371/journal.pcbi.1010571)
Supplement: S2 Appendix — (DOCX) [file pcbi.1010571.s002.docx]

S2 Appendix

Example Completed Design Planner Tables

This document contains examples of completed design planner tables, retrieved from <https://rr.peercommunityin.org/articles/all_recommended_articles> [02 May, 2022].

Pownall, M., Pennington, C. R., Norris, E., & Clark, K. (2021, September 29). Evaluating the pedagogical effectiveness of study preregistration in the undergraduate dissertation: A Registered Report. [Stage 1 Registered Report]. <https://osf.io/9hjbw> [1]

| **Research question** | **Hypotheses** | **Sampling plan** | **Analysis plan** | **Rationale for deciding the sensitivity of the test for confirming or disconfirming the hypothesis** | **Interpretation given different outcomes** | **Theory that could be shown wrong by the outcomes** |
| --- | --- | --- | --- | --- | --- | --- |
| 1. Is preregistration a useful pedagogic practice to improve students' perceived understanding of research methods and statistics in the undergraduate dissertation? | We generally predict that attitudes to statistics will improve over time as a result of engaging with the third-year dissertation process itself, but that preregistration will have an additive effect on this. Students in the preregistration group will show a marked improvement compared to those in the control (H1) | Two-hundred and forty final-year undergraduate Psychology students will be initially recruited with approximately 20% attrition expected at Time 2 based on prior research sampling from online platforms (Palan & Schitter, 2018). The final planned sample size is therefore 200 participants. See design and participants for power analysis in more detail. | 2 (Group: preregistration vs control) x 2 (Time: time 1 vs. time 2) mixed ANOVA with attitudes to statistics as the dependent variable. | Simulation based power analyses conducted using the superpower shiny package (Lakens & Caldwell, 2021) with 10,000 simulations indicate that this sample size will have 80% statistical power to detect an effect size of *np^2^* = .04 for the two-way interaction between Group and Time, and 80% power to detect small-moderate effects of *d* = .40 for the focal pairwise comparison between preregistration vs. control at Time 2 (Code/Output: <https://osf.io/y9vz7/>).  We will also run a sensitivity analysis to compare our achieved sample size with planned sample size (see Participants and design section for further details).  For our Bayesian analyses, we will adopt a F10 < 0.17 as evidence for the null, which is a conservative criteria for this analysis that will allow us to test support for the null or alternative hypothesis. | This could find that preregistration *does* impact students’ statistics attitudes, as we predict, or it could suggest that preregistration does not add benefits above and beyond differences that occur due to time (from time point 1 to time point 2).  No main effect of time would suggest that students do not change in their attitudes towards statistics as they progress through their academic studies in final year. However, our bayesian analyses will also reveal the *strength of evidence* we have to make these conclusions. | Theoretically, the notion that preregistration confers a tangible, pedagogical benefit to students in their dissertation process could be (un)supported by all of our proposed analyses.  Explanations for all results will be presented in the discussion. |
| 1. Does the process of preregistration enhance awareness and acceptance of questionable research practices (QRPs)? | We predict that preregistration will reduce acceptance of QRPs as ‘sensible’ for the preregistration compared to the control group (H2). |  | 2 (Group: reregistration vs control) x 2 (Time: time 1 vs. time 2) mixed ANOVA with acceptance of QRPs as the dependent variable. |  | Similarly, this analysis tests whether a preregistration process improves students’ awareness of QRPs; therefore, this analysis could find that preregistration *does* positively impact students’ awareness of QRPs, as we predict, or it could suggest that preregistration does not add benefits above and beyond differences that occur due to time (from time point 1 to time point 2). |  |
| 1. Does the process of preregistration improve perceived understanding of Open Science practices? | We predict that preregistration will improve perceived understanding of Open Science practices and terminology compared to the control group (H3). |  | 2 (Group: reregistration vs control) x 2 (Time: time 1 vs. time 2) mixed ANOVA with awareness of Open Science practices as the dependent variable. |  | As above, this analysis allows us to test whether preregistration improves students’ perceived understanding of Open Science practices. Similar to the above, a significant main effect of Group would indicate that preregistration does or does not impact students’ Open Science perceived understanding, independent from time effects.  Interactions of the ANOVA could find that preregistration *does* positively impact students' perceived understanding of Open Science, as we predict, or it could suggest that preregistration does not add benefits above and beyond differences that occur due to time (from time point 1 to time point 2). |  |
| 1. Do students recognise the benefits of the preregistration process in their undergraduate dissertation and are there any barriers/challenges to its implementation? | This research question is exploratory. We will first explore whether preregistration is associated with *Capability, Opportunity, and Motivation* (COM-B) *for preregistration* by comparing the preregistration. We will then conduct qualitative content analysis on participants’ free-text responses at Time 2. | This research question is exploratory and the same sample detailed above will be used to address this question. | A t-test comparing preregistration group vs control group at Time 1 with COM-B scores as the dependent variable.  Qualitative analysis using qualitative content analysis for free-text responses. | This research question is exploratory. Qualitative research typically does not share concerns of generalisability with quantitative research, so our planned sample size for this study will be sufficient for our qualitative research question, given the epistemological underpinnings of this approach. | This set of exploratory analyses allows us to test whether students have the sufficient capability, opportunity, and motivation to complete preregistration.  Qualitative analyses will shine light into whether students recognise any barriers or challenges, in order to provide more nuance to the quantitative analysis. |  |

Karhulahti, V-M., Vahlo, J, Martončik, M., Munukka, M., Koskimaa, R., von Bonsdorff, M. (2022, January 17). Identifying Gaming Disorders by Ontology: A Nationally Representative Registered Report. [Stage 1 Registered Report]. <https://osf.io/usj5b> [2]

| **RQ** | | **H** | | **Sampling** | | **Analysis** | | **Rationale** | | **Interpretation** | | **Theory** | |
| --- | --- | --- | --- | --- | --- | --- | --- | --- | --- | --- | --- | --- | --- |
| **A** | | **1a 1b 1c 1d** | | Nationally representative *N*=8000, which corresponds with the precision required to assess prevalence for 0.2 (*n*=7668). This prevalence is the lowest one found in our pilot (for GDT). | | We calculate prevalence rates and 95% confidence intervals with recommended cutoffs for the screening instruments. We apply the method described by Dienes: “If the CI lies mainly in the H0 interval and the remaining minority only in the grey interval, one could accept H0; similarly, if the CI lies mainly in the H1 interval and the remaining minority only in the grey interval, accept H1; otherwise more data are needed” (2021, p. 9). | | All instruments have a different ontological basis. Significant differences between prevalence rate proportions would be some evidence for these ontological bases representing different constructs. No difference would be evidence for these ontological bases representing the same construct. | | In each hypotheses, we set the H0 interval to the lower bound of the smallest obtained prevalence rate and the H1 interval to x2 of its upper bound. If the confidence intervals of the *differences between compared prevalence rates* fall mainly to the H1 area, we consider H1a–d corroborated. Null will be corroborated by the confidence intervals falling mainly to the H0 area. If the confidence interval falls mainly in the grey area between, none of the hypotheses are corroborated. | | H1 (if all sub hypotheses are corroborated) would support the position that the DSM-IV and/or Self-assessment based “gaming disorder” constructs are different from those of ICD-11 and/or DSM-5, prevalence-wise. H0 (if all sub hypotheses are refuted) would support the similarity of the constructs, respectively. Both types of evidence would be theoretically meaningful. In case of mixed findings, we do not draw full theoretical implications for H1 or H0. | |
| **B** | | **2a** | | N/A | | We test *P*(DSM-5\|ICD- 11)≈1 and *P*(DSM-  5+GDT\|DSM-IV)≈1. To  control Type 2 error, we allow variation by the lower bound of the binomial probability 50% confidence interval for the conditional probability *P*=1 (at n=16/16; 0.917, 1). For possible null testing, we set the binomial probability confidence interval at 95% for the conditional probability *P*=1 (n=16/16; 0.794, 1). | | All instruments have a different ontological basis, but previous literature has shown 100% of those who meet ICD-11 based criteria to also meet DSM-5 criteria. We thus expect the ICD-11 criteria meeting group to also meet the DSM-5 cutoff. To control Type  2 error, we allow variation based on the 50% confidence interval of *P*=1 (at n=16/16; 0.917, 1). | | If the obtained sample of ICD-11 identified participants meets *P*(ICD- 11\|DSM-5)>0.917, we consider H2a corroborated and this as evidence for the ICD-11 and DSM-5 based constructs to be similar by overlap. If the obtained sample of GDT- identified participants meets *P*(ICD-11\|DSM- 5)<0.794, we consider this as evidence for the ICD-11 and DSM-5 based constructs to be different by overlap. | | If both H2a and H2b are corroborated, this would be evidence for all ICD-11, DSM- 5, and DSM-IV based constructs to overlap. If H2a and H2b are not corroborated, this will be evidence for construct differences (three possible differences: ICD-11 vs DSM-5, ICD-11 vs DSM- IV, DSM-5 vs DSM-IV).  Similarities support the singular “gaming disorder” construct and differences support the multiplicity thereof. In case of mixed findings, we do not draw full theoretical implications for H2 or H0. | |
| **B** | | **2b** | |  |  |  |  | Our pilot indicates both ICD-11 and DSM-5  based groups to also meet DSM-IV criteria. We thus expect the ICD-11 and DSM-5 criteria meeting groups to also belong to the DSM-IV criteria meeting group (in the same way as above). | | [Same as above, with GDT, IGDT10, GAS7] | |  |  |
| **C** | | **3a** | | The sample size is based on a power analysis for the Welch t-test (one-sided). We expect that the size of the effect between the DSM-IV group and the rest of the sample will be approximately *d*=0.22–0.41 with GPH-2 and GMH-2. For the desired power level of 0.9, alpha set to 0.0125, and the expected sizes of the DSM-IV group (*n*=712 / 8.9%) and self- | | We compare the means of mental and physical health, measured by | | Based on pilot data, we expect the DSM-IV criteria meeting group to have lower general mental health than the remaining sample. Previous evidence and our pilot indicate the same for physical health, and we expect this, too. | | If either the mental or physical health of the DSM-IV based criteria meeting groups are significantly (d*≥*0.22 at alpha 0.025) lower than those of the rest of the sample, we consider H3a corroborated. A nonsignificant or below *d*=0.22 effect will not corroborate the H3a. | | If corroborated, we consider that as evidence for the THL1 (self-assessed) or GAS7 (DSM-IV pathological gambling based) criteria meeting groups to have lowered mental and/or | |
| **C** | | **3b** | | assessment group (*n*=192 / 2.4%) when the target sample size is *N*=8000, we have power to reliably detect *d*=0.138 and *d*=0.257,  respectively. Although the latter does not meet our lowest effect size of interest (*d*=0.22), we have reason to believe that our prevalence rates will be higher. If the self-assessment criteria meeting group will be smaller than the one needed to detect the observed effect, we will not make inferences in H3b unless the upper bound of the effect’s confidence interval does not exceeds our smallest effect size of interest (which would support uninteresting effect). | | GPH-2 and GMH-2, between the DSM_IV group and the rest of the sample (two-tailed ANOVA). This is repeated with self-assessment criteria. We carry out the Welch t-test twice with a corresponding alpha level 0.025 (multiple comparison correction). In case of nonsignificant results, we continue with equivalence testing (as below). | | [Same as above with mixed evidence for THL1] | | Same as above with THL1, where equivalence testing can support the null.] | | physical health (no causal interpretations). This would support the two instruments to measure one or more constructs, which are distinct health-wise in relation to the general population. Equivalence, in turn, would support equal health levels between those with “gaming disorder” in the two respective ontological domains and the general population. | |
| **C** | | **3c** | | N/A | | We use the TOSTER::TOSTone.ra w() function from the TOSTER package. First, we will compute raw means (mdiff1, mdiff2) and standard deviations (sddiff1, sddiff2) for both effects (differences between groups based on the GAS7 and THL1 classification). In TOSTER::TOSTone.ra  w() function *m* = 0, *sd =* (sddiff1, sddiff2)/2 and *mu* = mdiff1 – mdiff2. Lower and upper equivalence bound will be +- 0.2 *sd*. If we do not find equivalence, we move to test significance both ways. | | Based on pilot data, both equivalence and difference are possible. We set two competing hypotheses. | | We test significance between the health levels of the two groups and consider d*≥*0.22 (at alpha 0.025) evidence for difference. | | Corroborated H0 would weakly support self-assessed “gaming disorder” and DSM- IV based “gaming disorder” to represent the same or similar construct health-wise. If we find a difference, this would some evidence for the multiplicity regarding the two constructs health-wise. Fully corroborated H3/H0 would require all sub hypotheses to be corroborated. | |

**RQ**: How do screening instruments that derive from different ontological understandings differ in identifying GRHP groups?

**RQ-A**: How do GRHP screening instruments that derive from separate ontological understandings differ in *their prevalence rates (how many)*?

**RQ-B**: How do GRHP screening instruments that derive from separate ontological understandings differ in *who they identify (what characteristics)*?

**RQ-C**: How do GRHP screening instruments that derive from separate ontological understandings differ in the *health of their identified groups (how healthy)*?

**H1**: *We expect the ICD-11 and DSM-5 based GRHP prevalence rates to be meaningfully lower than the DSM-IV and Self-assessment based prevalence rates.*

**H1a**: *We expect the ICD-11 based prevalence rate to be meaningfully lower than the DSM-IV based prevalence rate.*

**H1b**: *We expect the ICD-11 based prevalence rate to be meaningfully lower than the Self-assessment based prevalence rate.*

**H1c**: *We expect the DSM-5 based prevalence rate to be meaningfully lower than the DSM-IV based prevalence rate.*

**H1d**: *We expect the DSM-5 based prevalence rate to be meaningfully lower than the Self- assessment based prevalence rate.*

Pennington, C. R., Monk, R. L., Heim, D., Rose, A. K., Gough, T., Clarke, R., Knibb, G., & Jones. A. (2022, January 25). To help or hinder: Do the labels and models used to describe problematic substance use influence public stigma? [Stage 1 Registered Report]. <https://osf.io/4vscg> [3]

| **Question** | **Hypothesis** | **Sampling plan** | **Analysis Plan** | **Rationale for deciding the sensitivity of the test for confirming or disconfirming the hypothesis** | **Interpretation given different outcomes** | **Theory that could be shown wrong by the outcomes** |
| --- | --- | --- | --- | --- | --- | --- |
| RQ1: Does the health condition of ‘drug use’ or ‘health concern’ influence public stigma and discrimination ? | The health condition of ‘drug use’ will elicit significantly greater stigma and discrimination compared to ‘health concern’. | A sample size of 1,578 participants will be recruited, allowing for >90% statistical power to conduct equivalence tests on the upper and lower equivalence bounds of −ΔL = -.20 and ΔU = .20 with a = .01.  Sensitivity power analyses will be conducted in the event we do not hit this target, indicating the effect size we were powered to detect. | Independent samples equivalence tests will be conducted on the between-participants factor of health condition, with the upper and lower equivalence bounds of −ΔL = -.20 and ΔU = .20. To allow direct comparisons with Kelly et al. (2021) and Rundle et al. (2021), these will be conducted on the five discrete subscales of the Stigma & Attribution Assessment and the total score from the Personal & Perceived Public Stigma Measure. We will also conduct these on the reward and punishment indices of the novel Financial Discrimination Task. | If the 99% CI lies outside of the equivalence region (−ΔL = -.20 and ΔU = .20), we will assert a meaningful effect. If the 99% CIs lie within the equivalence region, we will assert that we did not detect a meaningful effect (given the effect size that our sample is powered to detect). | If we find evidence of a meaningful effect, then this will provide support for previous findings suggesting that drug use is more stigmatised compared to other health conditions. If this effect is equivalent, then this will suggest that the effect was smaller than our effect size of interest. | Research indicates that problematic substance use is one of the most heavily stigmatised health conditions (Kilian et al., 2021; Room et al., 2001; Schomerus et al., 2011) with individuals diagnosed with a substance use disorder (SUD) routinely viewed as dangerous, unpredictable, helpless, and non-human (Dyregrov & Bruland-Selseng, 2020; Nieweglowski et al., 2017). If the findings of this analysis are equivalent, then this would suggest that future research is required to assess whether problematic substance use is heavily stigmatised compared to general health concerns and the effect sizes that are deemed meaningful within this research field. |
| RQ2: Does the aetiological label of ‘chronically relapsing brain disease’ or ‘problem’ influence public stigma and discrimination towards problematic substance use? | Non-directional: There is mixed evidence regarding whether the ‘disease’ label exacerbates or lessens stigma, and the findings may differ based on discrete elements of stigma measured, which we aim to test. | As above. | Independent samples equivalence tests will be conducted on the between-participants factor of aetiological label. This analysis will be conducted on the ‘drug use’ health condition only (as we are interested in examining stigma and discrimination towards problematic substance use). These will be conducted on the five discrete subscales of the Stigma & Attribution Assessment and the total score from the Personal & Perceived Public Stigma Measure. We will also conduct these on the reward and punishment indices of the novel Financial Discrimination Task. | As above. | If we find evidence of a meaningful effect, then this will provide support either for (lower stigma) or against (higher stigma) the brain disease model of addiction (BDMA). If this effect is equivalent then this will suggest that the effect was smaller than our effect size of interest.  If different effects are concluded from the two self-report questionnaires, then this will suggest that one of the measures may be better (more sensitive) for detecting public stigma compared to the other. Specifically, it is possible that the Stigma & Attribution Assessment (Kelly et al., 2021) will result in different findings to the Personal & Perceived Public Stigma Measure (Rundle et al., 2021) because the former assesses discrete elements of stigma whereas the latter is more general. This will inform future research of what measures may be best suited to examining stigma.  If this effect is only found for the discrimination measure, then this may suggest that indirect measures are better suited than self-reports, which are susceptible to socially desirable responses. | The mixed-blessings model (Haslam & Kvaale, 2015) suggests that the disease model may lower stigmatising perceptions of blame but decrease prognostic optimism (personal agency) and increase perceptions of danger and need for continuing care (see Kelly et al., 2021). If the findings from this analysis are equivalent, then future research would be required to test support for both the BDMA and the mixed-blessings model. This analysis will also provide evidence of which measures are best suited to measuring stigmatising perceptions towards problematic substance use. |
| RQ3: Does attributional judgement - high versus low treatment stability - influence public stigma and discrimination towards problematic substance use? | Non-directional: Neither Kelly et al. or Rundle et al. (2021) manipulated attributional judgement in their studies, but this is an additional factor that we recognised as a difference between the two. | As above. | Independent samples equivalence tests will be conducted on the between-participants factor of attributional judgement. This analysis will be conducted on the ‘drug use’ health condition only. These will be conducted on the five discrete subscales of the Stigma & Attribution Assessment and the total score from the Personal & Perceived Public Stigma Measure. We will also conduct these on the reward and punishment indices of the novel Financial Discrimination Task. | As above. | Previous research has not explicitly examined whether attributional judgement influences public stigma and discrimination towards problematic substance use. If we find evidence of a meaningful effect, then this would suggest that attributional judgement (low vs. high treatment stability) influences public stigma and/or discrimination. If this effect is equivalent, then it will suggest that the effect was smaller than our effect size of interest. | Previous research has shown that attributional judgement affects how individuals who use substances perceive themselves (self-image bias; Monk & Heim, 2013). However, to our knowledge, this has not been tested on public stigma and discrimination. |

Eder, A. B., Dignath, D., Gamer, M. (2022, February 08). Motivational Control of Habits: A Preregistered fMRI Study. [Stage 1 Registered Report]. <https://osf.io/k8ygb> [4]

| Question | Hypothesis | Sampling plan | Analysis Plan | Rationale for deciding the sensitivity of the test for confirming or disconfirming the hypothesis | Interpretation given different outcomes | Theory that could be shown wrong by the outcomes |
| --- | --- | --- | --- | --- | --- | --- |
| Is the insensitivity of cue-motivated action tendencies to posttraining changes in reward values an intrinsic design feature of habits (as proposed by dual-action psychologies) OR is it because the benefits of controlling “habitual” action tendencies do not outweigh the intrinsic costs of engaging in control (as suggested by expected value of control theory, EVC)? | According to EVC, cognitive control is intensified when the benefits of suppressing a dominant action tendency will justify the intrinsic costs of engaging in control. A central hub for these calculations on the neural level is the dorsal anterior cingulate cortex (dACC). In a Pavlovian-to-instrumental (PIT) transfer test, dACC activity should increase during presentations of Pavlovian cues associated with devalued outcomes relative to cues associated with non-devalued/neutral outcomes and in comparisons with PIT tests performed before the devaluation. | *N = 41*  A-priori power analysis for the detection of an increased dACC activation after relative to before the outcome devaluation in a one-tailed paired t-test with 1-β = 0.95 and α = .05. | GLM approach with Pavlovian Cue and Responses as regressors for neural activations in PIT tests before and after the devaluation treatment; follow-up analyses with t-test comparisons of activation differences before and after devaluation of the associated outcome (for details see Specification of fMRI Models for a Test of Brain Activity Hypotheses) | ES estimates obtained from a behavioural pilot study (*dz* = 0.55) and from a source study (Eder & Dignath, 2016b, Exp 2, *dz* = 0.53).  ES = mean difference in the magnitudes of behavioural PIT effects before and after outcome devaluation. | Increased dACC activity after outcome devaluation would be in line with the EVC model of dACC function in habit control. Finding no dACC effect, and/or observing activation differences in unrelated brain regions, would not support this model. | Increased dACC activity after devaluation of the outcome would support EVC theory and challenge dual action psychologies that claim a structural independence of habits from outcome representations. |
| Was the devaluation treatment effective? | Working (response rate) for the devalued outcome O1 should be lower in Transfer Test 2 after compared to before devaluation in Transfer Test 1 | *N = 41* | ES = difference between response rates (R1) in the first and second transfer tests.  (1) Comparison in a paired t-test.  (2) If no significant test result in (1), then TOST procedure for the rejection of SESOI dz = 0.40. | ES in the pilot study: dz = 1.1 (95%CI [0.58, 1.61]). Sufficient power (0.80) for the detection of d ≥ 0.40 in one-sample t-tests (TOST). | Significant test result in (1) would confirm that the devaluation treatment was effective.  Non-significant TOST result in (2) would indicate absence of a meaningful effect (= failure). | Manipulation check (behavioural data).  Failure would threaten the conclusiveness of the study: Report is published at OSF but will not be submitted to PCI for Stage 2 approval. |
| Was the procedure appropriate for generating cue-dependent (‘habitual’) action tendencies (Transfer Test 1)? | Pavlovian cues (CS1, CS2, CS3) should specifically increase numbers of keypresses that were associated with the same outcome (R1, R2, R3) relative to the baseline condition (with presentations of CS-). | *N = 41* | ES = specific PIT effect calculated for Transfer Test 1  (1) 2-way interaction effect between Pavlovian Cue and Instrumental Relation in a 4x3 rm-ANOVA  (2) If no significant test result in (1), then TOST procedure for the rejection of SESOI dz = 0.40. | ES in the pilot study: ηp2 = 0.238 (dz = 0.85, 95%CI [0.37, 1.31]). Sufficient power (0.80) for the detection of d ≥ 0.40 in one-sample t-tests (TOST). | Significant test result in (1) would confirm cue-dependent response tendencies in Test 1.  Non-significant TOST result in (2) would indicate absence of a meaningful effect (= failure). | Manipulation check (behavioural data).  Failure would threaten the conclusiveness of the results. Report is published at OSF but will not be submitted to PCI for Stage 2 approval. |
| Was the procedure appropriate for generating cue-dependent (‘habitual’) action tendencies (Transfer Test 2)? | Non-devalued Pavlovian cues (CS2, CS3) should specifically increase numbers of keypresses associated with the same outcome (R2, R3) relative to the baseline condition (with presentations of CS-). | *N = 41* | ES = specific PIT effect (R2, R3), calculated for Transfer Test 2  (1) 2-way interaction effect between Pavlovian Cue and Instrumental Relation in a 3x2 rm-ANOVA 2)  (2) If no significant test result in (1), then TOST procedure for the rejection of SESOI dz = 0.40. | ES in the pilot study: ηp2 = 0.43 (dz = 0.94, 95%CI [0.45, 1.42]). Sufficient power (0.80) for the detection of d ≥ 0.40 in one-sample t-tests (TOST). | Significant test result in (1) would confirm cue-dependent response tendencies in Test 2.  Non-significant TOST result in (2) would indicate absence of a meaningful effect (= failure). | Manipulation check (behavioural data).  Failure would seriously question the conclusiveness of the results.  Report is published at OSF but will not be submitted for Stage 2 approval to PCI. |

*Note.* ES = effect size; SESOI = minimum effect size of interest; TOST = two one-sided tests procedure; OSF = Open Science Framework

Lovell, M., Dienes, Z. (2022, February 14). Minimal mindfulness of the world as an active control for a full mindfulness of mental states intervention: A Registered Report and Pilot study. [Stage 1 Registered Report]. <https://osf.io/tx54k> [5]

| **Question** | **Hypothesis** | **Sampling plan** | **Analysis Plan** | **Rationale for deciding the sensitivity of the test for confirming or disconfirming the hypothesis** | **Interpretation given different possible outcomes** | **Theory that could be shown wrong by the outcomes** |
| --- | --- | --- | --- | --- | --- | --- |
| Do expectations to improve account for difference in outcomes? | Mindfulness of Mental States and Mindfulness of the World will have ‘equivalent’ expectations for a change in mindfulness and for depression and anxiety. | Estimated 120 participants needed. Sample until one of: i) *B* > 3; ii) *B* < 1/3; or iii) N = 300 | Separate analyses run for mindfulness and for depression/anxiety expectations. Bayes Factor of expectancy difference between Mental States and World group. As expectations should be limited to tracking outcome changes, H1 estimated at double the likely effect size (2x.2=.4) - and modelled as a normal distribution (M=.2,SD=.1) as larger, not smaller expectations likely to lead to larger outcomes. If expectations are non-equivalent but this difference is small, we will factor this out by adjusting the means for expectation effects. | *B* > 3 is the amount of evidence just worth taking note of, by tradition | *B* > 3, expectations non-equivalent, *B* < 1/3, expectations equivalent. | Any effects noticed in the mindfulness of mental states intervention are not due to the placebo effect. If expectations track outcome changes, the effect of the manipulation cannot be determined as separate from these expectation effects (manipulation check). |
| Did the time taken to complete the intervention affect the outcome obtained? (non-crucial) | Mindfulness of Mental States and Mindfulness of the World will take ‘equivalent’ amounts of time to complete. | Non-crucial test. | Bayes Factor difference in time taken between mental states and world group for using model of H1 with an SD of the raw difference in TMS scores between interventions divided by slope of regression of TMS on time taken to complete an intervention. | As above | *B* > 3, time taken non-equivalent, *B* < 1/3, time taken equivalent. | Any effect in outcome variables may be due to time taken to complete intervention (non-crucial manipulation check). |
| Has metacognitive task performance been equalised between conditions? | Mindfulness of Mental States will have ‘equivalent’ d’ scores to Mindfulness of the World. | Non-crucial test. | Bayes Factor of mean difference in d’ between groups, with H1 modelled as a half-normal with an SD of the difference in meta-d’ between groups | As above | *B* > 3, task performance non-equivalent, *B* < 1/3, task performance equivalent. | Any effect in meta-d’ may be due to task performance (non-crucial manipulation check). |
| Is mindfulness a metacognitive practice? | Mindfulness of Mental States will increase d’ adjusted meta-d’ scores over Mindfulness of the World. | Estimated 160 participants needed. Sample until one of: i) *B* > 3; ii) *B* < 1/3; or iii) N = 300 | Bayes Factor of interaction contrast between mental states group and world group by pre vs post, using model of H1 with SD =.5 meta-d’ units difference. | As above | *B* > 3, metacognition training increases mindfulness. *B* < 1/3, the extra metacognitive training is not useful for a short mindfulness intervention in order to increase mindfulness. | Metacognitive mindfulness training enhances adjusted metacognitive sensitivity (manipulation check). |
| Does training in mindfulness of mental states rather than the world promote more mindfulness of mental states? | Mindfulness of Mental States will increase TMS-D (decentring) scores over Mindfulness of the World. | Estimated 220 participants needed. Sample until one of: i) *B* > 3; ii) *B* < 1/3; or iii) N = 300 | Bayes Factor of interaction contrast between mental states group and world group by pre vs post, using model of H1 with SD =.2 Likert units difference. | As above | *B* > 3, metacognition training increases mindfulness. *B* < 1/3, the extra metacognitive training is not useful for a short mindfulness intervention in order to increase mindfulness. | Metacognitive mindfulness training enhances the facilitation of mindfulness of mental states in the short-term (manipulation check). |
| Does a metacognitive component of mindfulness account for its positive effects on mental health, specifically in reducing depression? | Mindfulness of Mental States will decrease PHQ-8 (depression), scores over Mindfulness of the World. | Estimated 180 participants needed. Sample until one of: i) *B* > 3; ii) *B* < 1/3; or iii) N = 300 | As above | As above | *B* > 3, metacognitive training is an important component of the effectiveness of mindfulness on depression in the short term. *B* < 1/3, metacognitive training is not an important component of short-term mindfulness interventions on depression. | Metacognitive training is key to the beneficial effects of short-term mindfulness interventions on depression. |
| Does a metacognitive component of mindfulness account for its positive effects on mental health, specifically in reducing anxiety? | Mindfulness of Mental States will decrease GAD-7 (anxiety), scores over Mindfulness of the World. | Estimated 220 participants needed. Sample until one of: i) *B* > 3; ii) *B* < 1/3; or iii) N = 300 | As above | As above | *B* > 3, metacognitive training is an important component of the effectiveness of mindfulness on mental health in the short term. *B* < 1/3, metacognitive training is not an important component of short-term mindfulness interventions on anxiety. | Metacognitive training is key to the beneficial effects of short-term mindfulness interventions on anxiety. |
| Is the Mindfulness of the World an effective mindfulness intervention? | TMS-D scores will increase in The Mindfulness of the World group over Waitlist controls | Estimated 180 participants needed. Sample until one of: i) *B* > 3; ii) *B* < 1/3; or iii) N = 300 | As above, except between World and Waitlist Group | As above | *B* > 3, Mindfulness of the World training increases mindfulness (TMS-D) scores, *B* < 1/3, the Mindfulness of the World group was not successful in increasing mindfulness scores. | Mindfulness of the world is an effective part of mindfulness training that can be targeted (manipulation check). |
| Mindfulness of the world has positive effects on mental health, specifically in reducing depression | Mindfulness of the World will decrease, and PHQ-8 (depression) scores compared to the Waitlist control. | Estimated 150 participants for PHQ-8 needed. Sample until one of: i) *B* > 3; ii) *B* < 1/3; or iii) N = 300 | As above | As above | *B* > 3, mindfulness of the world is an important component of the effectiveness of mindfulness on depression in the short term. *B* < 1/3, mindfulness of the world is not an important component of short-term mindfulness interventions on depression. | Mindfulness of the world is a component of the beneficial effects of short-term mindfulness interventions on depression. |
| Mindfulness of the world has positive effects on mental health, specifically in reducing anxiety | Mindfulness of the World will decrease, and GAD-7 (anxiety) scores compared to the Waitlist control. | Estimated 180 participants for GAD-7 needed. Sample until one of: i) *B* > 3; ii) *B* < 1/3; or iii) N = 300 | As above | As above | *B* > 3, mindfulness of the world is an important component of the effectiveness of mindfulness on mental health in the short term. *B* < 1/3, mindfulness of the world is not an important component of short-term mindfulness interventions on anxiety. | Mindfulness of the world is a component of the beneficial effects of short-term mindfulness interventions on anxiety. |

Szaszi, B., Palfi, B., Neszveda, G., Taka, A., Szecsi, P., Blattman, C., Jamison, J. C., Sheridan, M. (2022, February 15). Does alleviating poverty increase cognitive performance? Short- and long- term evidence from a randomized controlled trial. [Stage 1 Registered Report]. <https://osf.io/k56yv> [6]

| **Design Table Question** | **Hypothesis** | **Sampling plan** | **Analysis Plan** | **Interpretation given to different outcomes** |
| --- | --- | --- | --- | --- |
| Can a lump-sum unconditional cash treatment significantly improve the cognitive performance of the poor in the short-term? | We hypothesize that participants receiving unconditional lump-sum cash-transfers ($200) will show better cognitive performance (as measured with the general executive function index) in the short-term (2-5 weeks) compared to participants in the no treatment group. | Using a Bayesian Factor Design Analysis we found that the model provides correct inference in 82% and inconclusive inference in 18% of the simulations, while incorrect inferences would be made in less than 0.01% of the cases. | We will compare the cash and the no treatment groups, and conduct intention-to-treat Bayesian regression analysis. The parameters of the models are specified below:  Yi j = τ 1 Cash i + Xi λ + γ j + ε ij, where Y is the executive function index 2-5 weeks after the treatment, ‘Cash’ is dummy for the random assignment to the treatment involving Cash transfer, X contains the control characteristics, and γ is the fixed effect for each randomization block. | The statistical inferences and interpretation will be based on the Bayes Factors (BF). BF values above 10 and below 1/10 will be regarded as strong evidence for the alternative and the null hypothesis, otherwise we will interpret the BF as the strength of relative evidence between the hypotheses. |
| Can a lump-sum unconditional cash treatment significantly improve the cognitive performance of the poor in the long-term? | We hypothesize that participants receiving unconditional lump-sum cash-transfers ($200) will show better cognitive performance (as measured with the general executive function index) in the long-term (12-13 months) compared to participants in the no treatment group. | Using a Bayesian Factor Design Analysis we found that the model provides correct inference in 82% and inconclusive inference in 18% of the simulations, while incorrect inferences would be made in less than 0.01% of the cases | We will compare the cash and the no treatment groups, and conduct an intention-to-treat Bayesian regression analysis. The parameters of the models are specified below:  Yi j = τ 1 Cash i + Xi λ + γ j + ε ij, where Y is the executive function index 2-13 month after the treatment, ‘Cash’ is dummy for the random assignment to the treatment involving Cash transfer, X contains the control characteristics, and γ is the fixed effect for each randomization block. | The statistical inferences and interpretation will be based on the Bayes Factors (BF). BF values above 10 and below 1/10 will be regarded as strong evidence for the alternative and the null hypothesis, otherwise we will interpret the BF as the strength of relative evidence between the hypotheses. |

Breen. A. J., & Deffner, D. (2022, February 21). Investigating sex differences in learning in a range-expanding bird. [Stage 1 Registered Report]. <https://osf.io/v3wxb> [7]

| Question | Hypothesis | Sampling plan | Analysis Plan | Rationale for deciding the sensitivity of the test for confirming or disconfirming the  hypothesis | Interpretation given different outcomes | Theory that could be shown wrong by the outcomes |
| --- | --- | --- | --- | --- | --- | --- |
| Do learning ability and dispersal relate? | Sex differences in learning ability are related to sex differences in dispersal. | Use colour-reward reinforcement data from three study sites in great-tailed grackles—a species undergoing rapid range expansion, where males disperse. | Bayesian experience weighted attraction (EWA) model; modelling the influence of sex on two parameters of grackles’ colour- reward reinforcement learning: speed and sampling rate (where sampling is defined as switching between choice- options). | This method can* capture whether, and, if so, how multiple latent learning strategies simultaneously guide grackles’ decision making—an analytical advantage over more traditional methods (e.g., comparing trials to passing criterion) that ignore the potential for equifinality.  *We performed agent-based simulations to ensure our reasonable model-fit/effect detection *a priori*. | Hypothesis confirmed in full or in part:  Males are speedier and sample less than females *because range expansion disfavours slow, error-prone learning strategies in range expansion*.  Males are speedier than females *because range expansion disfavours slow learning strategies in range expansion*.  Males sample less than females *because range expansion disfavours error-prone learning strategies in range expansion*.  Between and/or within population differences exist with respect to any of the  above cases because range expansion results in a spatial sorting of learning ability  Hypothesis not confirmed:  Males and females do not differ in their colour-reward reinforcement learning; and, consequently, between or within population differences not detected because (not mutually exclusive) such sex- mediated differences never existed; too much time has passed since settlement and differences are no longer detectable as females ‘catch up’ (e.g., via assortative mating) and/or males ‘lose’ their learning ‘edge’ (e.g., via shifts in favourable post- establishment phenotypes) over successive breeding generations; our measure of learning ability does not capture this dynamic.  Females are faster and/or sample less than males because (not mutually exclusive) range expansion disfavours slow, error-prone learning strategies in females as they provide the bulk of parental care; females have ‘overtaken’ (e.g., via shifts in favourable post-establishment phenotypes) males across successive breeding generations. | N/A |
|  |  |  |  |  |  |  |

Balagtas, J. P. M., Tolomeo, S., Ragunath, B. L., Rigo, P., Bornstein, M. H., & Esposito, G. (2022, March 16). Neuroanatomical Correlates of System-justifying Ideologies: A Pre-registered Voxel-based Morphometry Study on Right-Wing Authoritarianism and Social Dominance Orientation. [Stage 1 Registered Report]. <https://osf.io/btkwq> [8]

| **Question** | **Hypothesis** | **Sampling Plan** | **Analysis Plan** | **Rationale for deciding the sensitivity of the test for confirming or disconfirming the hypothesis** | **Interpretation given different outcomes** | **Theory that could be shown wrong by the outcomes** |
| --- | --- | --- | --- | --- | --- | --- |
| In the social and political context of Singapore, are RWA and SDO scores correlated? | H1: RWA and SDO will be strongly correlated despite the low ideological contrast in Singapore’s sociopolitical system | One-tailed Correlation: Bivariate normal model  Alpha level, p = .05  N = 82 participants  Power = 0.99 to detect medium effect size, r = .30 | Pearson’s correlation test between RWA and SDO scores | The degree of RWA and SDO correlation is fairly predictable given the tenets of the Dual Process Model. That is, because Singapore is a country that is low in ideological contrast, we would expect a low correlation between SDO and RWA. However, the DPM does not account for top-down ideological influence, such as the institutionalised multiculturalism seen in Singapore. Taken together, we expect a significant positive correlation but since this specific correlation has not been documented in Singapore in previous works, we opted for a conservative but medium effect size. | H1 is supported if the test reveals a statistically significant correlation between RWA and SDO.  Significantly negative correlations would disconfirm the hypothesis. | Dual Process Model; if RWA and SDO are shown not to be correlated, this would follow the expected trend as predicted by DPM where RWA and SDO will be independent of one another in countries with low ideological contrast. This would suggest that multiculturalism either does not influence RWA and SDO in the same direction or not strongly enough to exceed the significance threshold. We may then rule out the possibility of multiculturalism playing an additional role to explain cross-cultural correlation between RWA and SDO. |
| Is/are there any overlapping region/s that are related to RWA and SDO? | H2: volume of the amygdala will be positively associated with both RWA and SDO scores | Linear multiple regression: Fixed model, R2 deviation from zero  Alpha level, p = .05  N = 82 participants  Power = 0.83 to detect medium effect size, f = .15 | ROI multiple regression analysis with RWA and SDO scores as independent variables regressed on mean GMV of amygdala. Age is included as a nuisance variable. | We decided on a medium effect size as this study is investigating a fairly new hypothesis. To the best of our knowledge, there has been no published work investigating brain structures that involve both SDO and RWA in a single study. Nonetheless, the correlation between amygdala volume and a conceptually similar scale (i.e. system justification scale) has been established in previous works. | H2 is supported if the test reveals both RWA and SDO have a statistically significant positive correlation with mean amygdala volume.  Insignificant correlation of either or both RWA and SDO with amygdala volume would disconfirm the hypothesis. Significantly negatively correlation of either or both RWA and SDO with amygdala volume would also disconfirm the hypothesis. | System-justification theory; if amygdala volume is not found to overlap with individual variation in RWA and SDO, there are at least two ways of interpreting this. One, there may be other regions that govern system-justifying ideologies in general. Two, individual variations in RWA and SDO do not lend themselves to comparable volumetric variation in neural substrates. In either case, this does not necessarily disprove SJT. |
| Is/are there any region/s that are related to RWA but not SDO? | H3: vmPFC volume will be negatively associated with only RWA | Linear multiple regression: Fixed model, R2 deviation from zero  Alpha level, p = .05  N = 82 participants  Power = 0.83 to detect medium effect size, f = .15 | ROI multiple regression analysis with RWA and SDO scores as independent variables regressed on mean GMV of vmPFC. Age is included as a nuisance variable. | We decided on a medium effect size as this study is investigating a fairly new hypothesis. To the best of our knowledge, there has been no published work investigating brain structures that involve both SDO and RWA in a single study. Granted previous work have not measured structural variation and simultaneously measured SDO scores, contributing work in brain lesion research and RWA scores point to this direction. | H3 is supported if the test reveals RWA, but not SDO, has a statistically significant negative correlation with mean vmPFC volume.  Insignificant correlation of both RWA and SDO with vmPFC volume would disconfirm the hypothesis. Significantly positive correlation of either or both RWA and SDO with vmPFC volume would also disconfirm the hypothesis. Significantly negative correlation of SDO with vmPFC would also disconfirm the hypothesis. | Dual Process Model; if vmPFC volumes are found to not correlate with RWA as predicted, similar interpretations can be made as with H2 (i.e. other brain regions governing RWA or vmPFC volume simply does not covary with RWA self-report scores); if vmPFC volume correlate with SDO but not RWA, this may suggest external factors may need to be accounted for in the DPM model, particularly, those that are pertinent to Singapore’s social and political context that may influence how RWA and SDO manifest. |
| Is/are there any region/s that are related to SDO but not RWA? | H4: left anterior insula volume will be negatively associated with only SDO | Linear multiple regression: Fixed model, R2 deviation from zero  Alpha level, p = .05  N = 82 participants  Power = 0.83 to detect medium effect size, f = .15 | ROI multiple regression analysis with RWA and SDO scores as independent variables regressed on mean GMV of left anterior insula, on separate analyses. Age is included as a nuisance variable. | We decided on a medium effect size as this study is investigating a fairly new hypothesis. To the best of our knowledge, there has been no published work investigating brain structures that involve both SDO and RWA in a single study. Though previous studies have only measured SDO in relation to fMRI-based research and do not account for RWA scores, neural activity in these areas associated with SDO aligns with a prediction of structural variation in these same brain regions. | H4 is supported if the test reveals SDO, but not RWA, have statistically significant negative correlations with mean left anterior insula volume.  Insignificant correlation of both RWA and SDO with left anterior insula volume would disconfirm the hypothesis. Significantly positive correlation of either or both RWA and SDO with left anterior insula volume would also disconfirm the hypothesis. Significantly negative correlation of RWA with left anterior insula would also disconfirm the hypothesis. | Dual Process Model; if left anterior insula volume is found to not correlate with SDO as predicted, similar interpretations can be made as with H2 (i.e. other brain regions governing SDO or left anterior insula volume simply do not covary with SDO self-report scores); if left anterior insula volume correlate with RWA but not SDO, this may suggest external factors may need to be accounted for in the DPM model, particularly, those that are pertinent to Singapore’s social and political context that may influence how RWA and SDO manifest. |

Xiao, L. Y. (2022, April 07). Breaking Ban: Assessing the effectiveness of Belgium’s gambling law regulation of video game loot boxes. [Stage 1 Registered Report]. <https://osf.io/5mxp6> [9]

| Question | Hypothesis | Sampling plan | Analysis Plan | Rationale for deciding the sensitivity of the test for confirming or disconfirming the hypothesis | Interpretation given different outcomes | Theory that could be shown wrong by the outcomes |
| --- | --- | --- | --- | --- | --- | --- |
| RQ 1: Has the Belgian ban succeeded in eliminating paid loot boxes from mobile games? | Hypothesis 1: None of the 100 highest- grossing iPhone games in Belgium will contain paid loot boxes. | 100 highest- grossing iPhone games in Belgium, following the convention established by the prior literature and with due consideration to a prior power analysis for H3, which is the central research question of the present study. | Hypothesis 1 will be accepted if zero, one, or two of the 100 highest- grossing games that will be coded contain paid loot boxes. | Absolute null is not optimal, as pointed out by peer- reviewers. 2% of type 1 error control will be included to account for potential false positives. | If >2%, then companies have not achieved near- perfect compliance with the measure. Companies should be instructed to better comply with the law. The gambling regulator should do more to enforce the law. (Note here that the interpretation will note the limitation that some of the companies might have a gambling licence: a point that will be put to the Belgian gambling regulator for confirmation.) | A legal ‘ban’ on paid loot boxes will mean that no paid loot boxes are available for purchase through regular channels that a normal player would encounter. |
|  |  |  |  |  | If ≤2%, then companies have achieved near- perfect compliance with the measure.  Companies should be commended for |  |

|  |  |  |  |  | complying with the law. The gambling regulator should also be  commended. |  |
| --- | --- | --- | --- | --- | --- | --- |
|  | Hypothesis 2: None of the games within the 100 highest- grossing iPhone games in Belgium that received an Apple Age Rating of 4+, 9+, or 12+ (i.e., not 17+) will contain paid loot boxes. | 100 highest- grossing iPhone games in Belgium that received an Apple Age Rating of 4+, 9+, or 12+  (i.e., not 17+) | Hypothesis 2 will be accepted if zero, one, or two of the games, within the 100 highest- grossing games that will be coded, that received an Apple Age Rating of 4+, 9+, or 12+ (i.e., not  17+) contain paid loot boxes. | Absolute null is not optimal, as pointed out by peer- reviewers. 2% of type 1 error control will be included to account for potential false positives. | If >2%, then companies have not achieved near- perfect compliance with the measure. Companies should be instructed to better comply with the law. The gambling regulator should do more to enforce the law.  More should be done particularly in relation to children. | A legal ‘ban’ on paid loot boxes will mean that no paid loot boxes are available for purchase by children through regular channels that a normal player would encounter. |
|  |  |  |  |  | If ≤2%, then companies have achieved near- perfect compliance with the measure. Companies should be commended for complying with the law. The gambling regulator should also be commended. |  |
| RQ 2: Has the | Hypothesis 3: Of | 100 highest- | Hypothesis 3 will | The assumed | If the Belgian loot | A legal ‘ban’ on |
| Belgian ban on paid | the highest- | grossing iPhone | be tested using a | hypothetical 65.0% | box prevalence rate | paid loot boxes will |
| loot boxes been | grossing iPhone | games in Belgium | binomial test (two- | paid loot box | is significantly | reduce the loot box |
| effective? | games, fewer will |  | sided test, p = .05) | prevalence rate was | **higher** than 65.0%, | prevalence rate of |
|  | contain paid loot |  | to identify whether | determined based | then the | that country. |
|  | boxes in Belgium |  | the percentage of | on a holistic | interpretation is |  |
|  | than in countries |  | the 100 highest- | overview of | that the measure |  |

|  | that have not banned loot boxes. |  | grossing iPhone games containing loot boxes in Belgium that will be found by the present study will be significantly different from a hypothetical loot box prevalence rate of 65.0%, which a Western country that has not restricted loot box sales is assumed to have. | previously found loot box prevalence rates in the UK, Australia, and China, as detailed in the manuscript. | has been **ineffective**, with limitations clearly noted.  If the Belgian loot box prevalence rate is significantly **lower** than 65.0%, then the interpretation is that the measure has been **effective**, with limitations clearly noted.  Comments as to the  degree of effectiveness will depend on the results of RQ 4. |  |
| --- | --- | --- | --- | --- | --- | --- |
|  |  |  |  |  | If the Belgian loot box prevalence rate is **not significantly different**, then the study will say that **no sufficent evidence** as to effectiveness has been found.  Alternative methods of potentially assessing effectiveness will be noted. |  |
| RQ 3: Is it possible | Hypothesis 4: UK | Three highly | Gameplay and | Practical and | Hypothesis 4 will | A player cannot |
| for a player to | iPhone games | popular and top- | examination of the | logistical | be accepted, if loot | **easily** circumvent a |
| circumvent the  Belgian ban on paid | known to contain  paid loot boxes will | grossing games,  published by | relevant games  under various | considerations. | box purchase is possible within one | legal ‘ban’ on paid  loot boxes. |

| loot boxes and | continue to offer | companies from | circumstances, as |  | or more of the |  |
| --- | --- | --- | --- | --- | --- | --- |
| purchase them | them for sale even | three different | detailed in the |  | games using any of |  |
| from within the | when the phone is | regions of the | manuscript. |  | the |  |
| country? | within | world, known to |  |  | abovementioned |  |
|  | geographical and | contain paid loot |  |  | methods. The |  |
|  | jurisdictional | boxes and in which |  |  | interpretation will |  |
|  | Belgium. | paid loot boxes |  |  | be that the law can |  |
|  |  | play a central role |  |  | be easily |  |
|  |  | in gameplay and |  |  | circumvented by |  |
|  |  | monetisation: |  |  | dedicated players; |  |
|  |  | Hearthstone, Brawl |  |  | the Belgian Gaming |  |
|  |  | Stars and Genshin |  |  | Commission should |  |
|  |  | Impact. |  |  | therefore consider |  |
|  |  |  |  |  | ways to force video |  |
|  |  |  |  |  | game companies to |  |
|  |  |  |  |  | better enforce |  |
|  |  |  |  |  | compliance with |  |
|  |  |  |  |  | the law. However, |  |
|  |  |  |  |  | if loot box purchase |  |
|  |  |  |  |  | is not possible |  |
|  |  |  |  |  | within one or more |  |
|  |  |  |  |  | of the games using |  |
|  |  |  |  |  | any of the |  |
|  |  |  |  |  | abovementioned |  |
|  |  |  |  |  | methods, the |  |
|  |  |  |  |  | interpretation is |  |
|  |  |  |  |  | that the law could |  |
|  |  |  |  |  | not be |  |
|  |  |  |  |  | circumvented in the |  |
|  |  |  |  |  | simple ways that |  |
|  |  |  |  |  | have been |  |
|  |  |  |  |  | attempted, |  |
|  |  |  |  |  | although other |  |
|  |  |  |  |  | potential |  |
|  |  |  |  |  | circumventions |  |
|  |  |  |  |  | remain untested |  |
|  |  |  |  |  | and possible. The |  |
|  |  |  |  |  | present study will |  |
|  |  |  |  |  | conclude that |  |
|  |  |  |  |  | companies might |  |

|  |  |  |  |  | have taken some technological measures to prevent circumventions of the Belgian ban, although further evidence would be required to confirm this (e.g., contacting the relevant company to request for confirmation of the compliance actions that have been taken). |  |
| --- | --- | --- | --- | --- | --- | --- |

Zhang, Q., Masuda, Y., Ueda, K., Toda, K., Yamada, Y. (2022, April 19). Is the past farther than the future? A registered replication and test of the time-expansion hypothesis based on the filling rate of duration. [Stage 1 Registered Report]. <https://osf.io/d9ec3>

[10]

| **Question** | **Hypothesis** | **Sampling plan** | **Analysis Plan** | **Rationale for deciding the sensitivity of the test for confirming or disconfirming the hypothesis** | **Interpretation given different outcomes** | **Theory that could be shown wrong by the outcomes** |
| --- | --- | --- | --- | --- | --- | --- |
| Q1: Which is psychologically closer, the future or the past? | H1: People will feel the past as farther than the future as in the original study (Caruso et al., 2013). | 936 participants will be recruited in Study 1.  Another 1308 participants will be recruited in Study 2.  The number of participants is based on power analyses. | Similar to Caruso et al.’s (2013) Study 1a and 1b, we will use a two-sample *t*-test to compare the estimated psychological distance in the past and future conditions in a between-subject design. | Power analysis conducted using G*Power 3.1 (Faul, Erdfelder, Lang, & Buchner, 2007) and pwr package 1.3-0 (Chamely, 2020) on R 4.0.5 (R Core Team, 2021) indicates that this sample size will have 95% statistical power to detect an effect size of *d* = 0.26 (*d* = 0.22 in Study 2) of the comparison between past group and future group in Study 1 at a significance level of .02. | In Study 1 (or 2), a significant difference between the past and the future condition (α = .02) indicates the acceptance of H1 in 1 month (or 1 year) condition, and the replication success of Caruso et al.’s (2013) Study 1a (or 1b). If H1 is not supported, there may be two reasons for it.  First, the results of psychological distance may be affected by the recruitment method of participants (i.e., crowdsourcing).  Second, there is a possibility that the TDE does not exist, or only exists under very limited conditions. | Q1 and H1 aim to replicate Caruso et al.’s (2013) study directly. Therefore, no theory could be proved wrong by the outcomes. |
| Q2-1: Which duration is fuller, the future or the past? | H2-1: Duration will be fuller in the past than in the future. | Using two different scales to measure the filling rate of duration (see Independent variables for details), participants will be divided into two equal groups, with 468 participants in each group for Study 1 and 645 participants in each group for Study 2. | We will use a paired *t-*test to compare whether the filling rate of duration differs in the past and future conditions in a within- subject design. | For H2-1 and H2-2, we also conducted an a priori sensitivity power analysis and found that the effect size is much smaller than *dz* = 0.4 (*r* = .2), which we were determined to use. Further, we will also use the full sample size to test these hypotheses.  (see Sample size and power analysis section for further details) | Significant difference between the past and the future condition (α = .02) indicates the acceptance of H2-1.  Significant positive correlation between psychological distance and the filling rate of duration indicates the acceptance of H2-2.  If H2-1 and H2-2 are not supported, it would suggest that the filling rate of duration is not an appropriate explanation for the TDE. If H2-2 is supported but H2-1 is not, we cannot explain TDE by the filling rate of duration. However, there might be some distortion in time estimation, which is based on the FDI-like effect, or the scale we use might not be valid enough for the filling rate of duration.  . | We are not aiming to contradict Caruso et al.’s (2013) explanations. Our aim is to provide another explanation for it, regardless of spatial-temporal metaphor. |
| Q2-2: Does the filling rate of duration affect the psychological distance in the future and past conditions? | H2-2: When the duration is fuller, the psychological distance will be farther. |  | We will conduct a correlation analysis between psychological distance and filling the rate of duration, using Spearman’s rank correlation coefficient. We will analyze both future and past data simultaneously. |  |  |  |

Jagini, K. K., & Sunny, M. M. (2022, April 26). Do task-irrelevant cross-modal statistical regularities induce distractor suppression in visual search? [Stage 1 Registered Report]. <https://osf.io/qjbmg> [11]

| **Question** | **Hypothesis** | **Sampling Plan** | **Analysis Plan** | **Interpretation n given different outcomes** |
| --- | --- | --- | --- | --- |
| Do task- irrelevant cross- modal (auditory) spatial regularities induce distractor suppression in visual search? (Experiment 1) | The response times (RTs) are expected to be shorter for HpValD “high- probability valid distractor location” trials compared to the HpInValD “high- probability invalid distractor location” trials. | We aim to recruit a minimum of 121 participants (who meets the participant selection criteria) from the Indian Institute of Technology.  **Sample Size Justification:**  In a previous study that is similar to the current experiments, Failing et al. (2019) reported an effect size of d = 0.602 by taking a difference between colour-match and colour-mismatch trials at two high probability distractor locations. Relying on the effect size from the previous study at the face value for an a priori power analysis is not recommended, as this might lead to underpowered studies (Dienes, 2021; Perugini et al., 2014). To guard against the underpowered study, we determined the smallest effect size of interest as the lower limit of 80% confidence interval for the effect size by following the advice of Perugini et al. (2014).  The determined effect size of interest is  0.332 (estimated using Shiny R web app: https://designingexperiments.shinyapps.io/ci_smd/  ). Conducting an a priori power analysis with effect size d = 0.332, given alpha = 0.02 and power ζ 90, yields a minimum of 121 participants required for each proposed experiment in a two-tailed matched- sample t-test (calculated using G*Power 3.1). This sample size is considerably larger than the typical experiments conducted using the additional singleton tasks (an average of around 26 participants in (Failing, Feldmann-Wüstefeld, et al., 2019; Wang & Theeuwes, 2018a, 2018b, 2018c)). | We will use paired t-test to compare experiment al conditions of *HpValD* (“high- probability valid distractor location”) with *HpInValD* “high- probability invalid distractor location”) conditions. Significanc e level – alpha set to 0.02), with power  >0.90. | If the RTs are significantly shorter for the HpValD condition than the HpInValD conditions, we claim the hypothesis 1. Otherwise, we will claim that the auditory spatial statistical regularities do not have influence on the distractor suppression in visual search tasks. |

| Do task- irrelevant cross- modal (auditory) non- spatial, frequency- based regularities induce distractor suppression in visual search? (Experiment 2) | The response times (RTs)are expected to be shorter for HpValD “high- probability valid distractor location” trials compared to the HpInValD “high- probability invalid distractor location” trials. | As above | As above | If the RTs are shorter for the HpValD condition than the HpInValD conditions, we claim the hypothesis 1. Otherwise, we will claim that the auditory non- spatial and frequency based statistical regularities do not have influence on the distractor suppression in visual search tasks. |
| --- | --- | --- | --- | --- |
| Do participants have awareness about the relationship between auditory (spatial) and visual distractor location regularities? (Experiment 1) | We hypothesis e that if the participant s are aware of the relationship p between auditory and visual distractor location regularities we expect that the score received by each location linearly decreases from its distance from the actual HpValD location. | Minimum of 75 participants.  **Sample Size Justification:**  Recent studies indicated that using a confidence rating scale and ranking methods are, arguably, more sensitive measures for testing awareness (Giménez-Fernández et al., 2020; Vadillo et al., 2020). Utilizing these sensitive measures to test awareness of statistical regularities in probabilistic cuing search tasks, the Vadillo et al. (2020) study indicated that participants are not unaware of the statistical regularities. Their study reported an effect size of Cohen's h = 0.57 for their meta-analysis of experiment 1 and 2. However, choosing the effect size from a previous study at the face value for an a priori power analysis is not recommended, as this leads to underpowered studies (Dienes, 2021; Perugini et al., 2014). To guard against the underpowered study, we determined the smallest effect size of interest as the lower limit of 80% confidence interval for the effect size by following the advice of Perugini et al. (2014).  The determined effect size of interest is 0.426 (estimated using Shiny R web app: <https://designingexperiments.shinyapps.io/ci_smd/>).The effect size of d = 0.426 requires a minimum of 75 participants for each proposed experiment to get power ≥ 90% with alpha set to 0.02 (calculated using G*Power 3.1) in a two-tailed matched-sample t-test. | We will use a linear mixed- effects model with random intercept for participants to predict a relationship between the scores received by each location from its distance from the HpValD location. | We will claim that the participants are aware of statistical regularities if the scores received by each location linearly decreases from its distance from the actual HpValD location.  Otherwise, we will claim that participants are unaware of statistical regularities. |
| Do participant s have awareness about the relationship between auditory (non- spatial and frequency based) and visual distractor location regularities? (Experiment 2) | As above | As above | As above | As above |

**References**

1. Pownall M, Pennington CR, Norris E, Clark K. Evaluating the pedagogical effectiveness of study preregistration in the undergraduate dissertation: A Registered Report. [Stage 1 Registered Report]. 2021 [cited 2022 May 13]. Available from: <https://osf.io/9hjbw>

2. Karhulahti V-M, Vahlo J Martončik M, Munukka M, Koskimaa R, von Bonsdorff M. Identifying Gaming Disorders by Ontology: A Nationally Representative Registered Report. [Stage 1 Registered Report]. 2022 [cited 2022 May 13]. Available from: <https://osf.io/usj5b>

3. Pennington CR, Monk RL, Heim D, Rose AK, Gough T, et al. To help or hinder: Do the labels and models used to describe problematic substance use influence public stigma? [Stage 1 Registered Report]. 2022 [cited 2022 May 13]. Available from: <https://osf.io/4vscg>

4. Eder AB, Dignath D, Gamer M. Motivational Control of Habits: A Preregistered fMRI Study. [Stage 1 Registered Report]. 2022 [cited 2022 May 13]. Available from: <https://osf.io/k8ygb>

5. Lovell M, Dienes Z. Minimal mindfulness of the world as an active control for a full mindfulness of mental states intervention: A Registered Report and Pilot study. [Stage 1 Registered Report]. 2022 [cited 2022 May 13]. Available from: <https://osf.io/tx54k>

6. Szaszi B, Palfi B, Neszveda G, Taka A, Szecsi P, Blattman C, et al. Does alleviating poverty increase cognitive performance? Short- and long- term evidence from a randomized controlled trial. [Stage 1 Registered Report]. 2022 [cited 2022 May 13]. Available from: <https://osf.io/k56yv>

7. Breen AJ, Deffner D. Investigating sex differences in learning in a range-expanding bird. [Stage 1 Registered Report]. 2022 [cited 2022 May 13]. Available from: <https://osf.io/v3wxb>

8. Balagtas JPM, Tolomeo S, Ragunath BL, Rigo P, Bornstein MH, Esposito G. Neuroanatomical Correlates of System-justifying Ideologies: A Pre-registered Voxel-based Morphometry Study on Right-Wing Authoritarianism and Social Dominance Orientation. [Stage 1 Registered Report]. 2022 [cited 2022 May 13]. Available from: <https://osf>.io/btkwq

9. Xiao LY. Breaking Ban: Assessing the effectiveness of Belgium’s gambling law regulation of video game loot boxes. [Stage 1 Registered Report]. 2022 [cited 2022 May 13]. Available from: <https://osf.io/5mxp6>

10. Zhang Q, Masuda Y, Ueda, K, Toda K, Yamada Y. Is the past farther than the future? A registered replication and test of the time-expansion hypothesis based on the filling rate of duration. [Stage 1 Registered Report]. 2022 [cited 2022 May 13]. Available from: <https://osf.io/d9ec3>

11. Jagini KK, Sunny MM. Do task-irrelevant cross-modal statistical regularities induce distractor suppression in visual search? [Stage 1 Registered Report]. 2022 [cited 2022 May 13]. Available from: <https://osf.io/qjbmg>
